# The Basic Determinants of Malnutrition: Resources, Structures, Ideas and Power

**DOI:** 10.34172/ijhpm.2020.259

**Published:** 2020-12-26

**Authors:** Jody Harris, Nicholas Nisbett

**Affiliations:** Institute of Development Studies, University of Sussex, Brighton, UK.

**Keywords:** Malnutrition, Basic Determinants, Resources, Structures, Ideas, Power

## Abstract

**Background:** While child undernutrition is improving overall, different population groups are experiencing different outcomes. What sets some groups apart is their experience of the ‘basic determinants’ of malnutrition, that underpin the ‘immediate’ and ‘underlying’ determinants, and that have been much less studied, defined and understood.

**Methods:** We undertook a qualitative narrative review based in two sets of ideas: nutrition’s basic determinants as laid out in the original United Nations Children’s Fund (UNICEF) framework, and critical concepts emerging from development studies. These ideas informed searches in Google Scholar, and resulting papers formed the basis for the review.

**Results:** Based on this literature, we expand and clarify the terminology of ‘basic determinants’ into a new framework, to include (1) resources and (material, human, social and natural) capitals at the basic level; (2) structures including social, market, legal and political systems driven by long-term demographic, economic, and environmental trends; and (3) ideas, beliefs and ideologies prevailing within a given society – crystallising into social norms and institutions – fundamentally shaping how societies are structured around power and marginalisation. We then illustrate with existing literature how these basic factors play out in the food, health and care determinants of malnutrition; and how theories of human rights and collective commons point us towards practical redressal options through improved participation and accountability.

**Conclusion:** We show here that the basic determinants are not a black box of ‘context,’ but can be broken down into comprehensible issues that are amenable to change, and should be considered explicitly in research and action to reduce the global burden of malnutrition.

## Introduction

The United Nations Children’s Fund (UNICEF) framework on the “causes of malnutrition and death” famously sets out factors at immediate, underlying and basic levels which shape outcomes for child nutrition.^1^ These include the relationship between nutrient intake and immunity at the immediate level, which is underpinned in turn by access to food and health systems, adequate childcare, and sanitary environments at the underlying level. These food, health and care aspects have been well described and evidenced, not only in the original UNICEF paper but in a large literature summarised in the most recent Lancet series on nutrition.^2^ It is the basic determinants at the base of the framework – which cover a range of social, economic and political drivers affecting malnutrition in all its forms – that have been much less studied, defined and understood. This paper offers a structure for understanding the complex mix of factors that make up the basic determinants. We review literature from multiple fields and disciplines, shaped from within our background in development studies and its allied disciplines such as geography, anthropology, political science and development economics. Apart from economics, these are literatures with which the field of nutrition has limited engagement, but which provide important evidence on the drivers of malnutrition, as well as suggesting options for addressing the basic determinants of malnutrition in practice.

## Research Approach

Taking the UNICEF framework as our starting point, we draw on multiple perspectives from the academic field of Development Studies to inform a conceptual structure for understanding the basic determinants of malnutrition. We then build on a narrative review of relevant literature to illustrate these determinants with empirical examples. The low- and middle-income countries that were the focus of the UNICEF framework now suffer from multiple forms of malnutrition (ie, including undenutrition and obesity/overweight)^3^ but the UNICEF framework is relevant given that many of the drivers at a basic or structural level, are the same or similar.^4^ We focus here on *child* malnutrition, as this was the focus of the UNICEF framework, but note the importance of understanding the drives of malnutrition over the lifecourse, including via links to maternal nutrition (though not exclusively so)^2,5,6^; and malnutrition as a vector of intergenerational poverty and exclusion.^7^

While there are no established guidelines for qualitative narrative reviews,^8^ the searches and synthesis here aimed to be systematic without being exhaustive. We set out to combine two sets of ideas: (1) An initial examination of previous iterations of the UNICEF malnutrition framework provided a list of the components that have historically been associated with the basic determinants. (2) At the same time, our previous engagement with literature and theory on the structural determinants of other relevant development issues (particularly food, health, and gender) provided a set of ideas less applied in international nutrition research. These two sets of ideas – nutrition’s basic determinants, and ‘big ideas’ in development studies – then formed the basis for literature searches in order to find research that could inform a more robust understanding of the basic determinants of malnutrition. The search terms are listed in Box 1 and were undertaken using the ‘advanced search’ function in Google Scholar, with no date, methodological or country limitations. As with the original UNICEF framework, our focus is largely on low- and middle-income countries as the historic focus of development studies and of international nutrition (though this is changing with a ‘universal development’ lens^9^ and the globalisation of food systems and cultures).^10^

**Box 1. Search Terms for Literature Searches**

- “Social determinants of health”
- “Health equity”
- “Structural violence” AND health
- “Food systems” AND equity
- Gender AND nutrition
- Women AND nutrition
- “Social exclusion” AND nutrition
- Marginalisation AND nutrition
- Power AND nutrition
- Politics AND nutrition
- Participation AND nutrition
- Accountability AND nutrition

Literature identified through these searches was screened for (1) relevance to understanding nutrition’s determinants; (2) relevance to child nutrition; and (3) level of citation (to get a sense of the centrality of papers to a body of literature). This process drew on the expertise of the authors and of key development studies colleagues working in the fields of gender studies, health equity, and participation and accountability through conversations during the process of this work. The most relevant and cited literature was narratively reviewed (first summarising the key theses of individual papers, then looking across thematic sets of papers to synthesise their core ideas), and the reviews used to populate the updated basic determinants framework. This work was then used as a set of examples illustrating how the basic determinants play out in the areas of food, health and care.

Certainly there are limitations to our approach to reviewing the literature, including the narrowness of our search term ‘nutrition’ (which would miss papers on related issues such as diets or perhaps *mal*nutrition), and our choice of development studies topics (which extend far beyond this list, but were chosen for their relevance to the original ‘basic determinants’ descriptions in the UNICEF framework). Rather than comprehensive review, our aim was to gather key ideas in the field of development studies through a review of papers central to development studies theory, and use these to expand and deepen the notion of the ‘basic determinants’ for a nutrition audience. A further limitation of the paper is that the literature on low- and middle-income countries is still largely focused on undernutrition, even though there is a smaller (but growing) number of papers on obesity and overweight and related burdens of non-communicable diseases. While this scope is necessarily limited, we also note that ‘malnutrition in all its forms’ is a problem now besetting countries in all income categories – while we focus less on higher income countries and draw less on the obesity/non-communicable diseases literature, there is still much of relevance here to those interested in the shared, basic drivers of malnutrition.

## Describing the Basic Determinants

The original UNICEF framework^1^ has stood the test of time because it was thoughtful about the ways it depicted the causes of malnutrition at multiple levels. In describing the basic determinants of nutrition, it focused on the political, ideological and economic structures that sit between the potential resources for households to achieve good nutrition and what is actually realised; and so arguably this approach takes a social lens to the basic determinants. In the most recent academic iteration of the framework,^11^ the description of basic determinants focuses on creating an enabling policy environment; this approach takes a governance or institutional lens to the basic determinants. UNICEF’s own update to the framework describes good governance, positive norms, and sufficient resources as underpinning the rest of the framework,^12^ building on our review below. Building on these iterations and the broader literature from our review, we conceptualise the basic determinants of nutrition as comprising three interrelated factors, described below.

###  Factor 1. Resources at the Basic Level

The resources, capacities and forms of capital available to people are tangible and intangible properties which shape the potential of individuals and groups to do things such as purchase or produce food, access proper healthcare, or care for family members (so to realise the immediate and underlying determinants which help achieve good nutrition). Traditional forms of material or financial capital including land and wealth resources have been long-studied in nutrition, with those in poverty known to be more vulnerable to poor nutrition.^13^ Beyond wealth, individuals and groups can improve their access to food, health and care by drawing on their human or ‘cultural’ capital, such as education or understanding of social communication; social and symbolic capital in terms of connections to family and broader networks and social standing^14^; and natural capital in the form of biodiversity and other ecosystem services.^15^

 This web of material, social, human and natural resources is in turn conditioned by how individuals, families or wider groups are seen within society (factor 3 below) and is mediated by the structures and drivers shaping society (factor 2).

###  Factor 2. Structures Shaping Society

Broad structures and drivers affecting access to resources and forms of capital are the existing social, market, legal and political institutions shaping society, in turn driven by long-term demographic, economic, and environmental trends. These condition the provision of goods and services relating to nutrition (such as food, care or clean water) which are accessed differently by different populations. These drivers change over time, and consequently the terrain on which people build their lives can shift. For instance, changes from traditional or feudal societies to industrial and mostly capitalist societies has profoundly influenced food production in a variety of different political systems^16^; globalization has accelerated the connectedness of markets, people and cultures via increased economic reach and migration^17^; urbanization has created groups with different needs in terms of working patterns and food requirements, and hence new social classes and political constituencies^18^; and environmental change in weather patterns and climate change has driven vulnerability of marginal populations.^19^

 The impact of such drivers on different people will depend on their existing web of resources and capitals (factor 1 above) while their exposure to these drivers will depend on their position within society and their access to political and social institutions of relevance, which are structured by normative and ideological forces (factor 3 below).

###  Factor 3. Forces Underlying Social Trends

The ideas, beliefs and ideologies prevailing within a given society fundamentally shape how that society is structured.^20^ Beliefs relating to the relative standing of different groups (based on issues such as gender, ethnicity, religion, age, disability and sexuality) produces stigma, marginalization and inequity for certain groups,^21^ and these different dimensions interact to condition access to the resources necessary for achieving good nutrition, as well as shaping power imbalances among different groups in their control over social and political processes.^22^ These power imbalances can occur at micro level, determining local entitlements to goods and services, or macro level, conditioning voice and representation in broader political decision-making.^23^

 It is the crystallisation of ideas and beliefs into social norms, such as the role of women or the dominance of certain religions, and into political and social institutions, such as policies and legal systems (factor 2 above), which in turn shape the resources and forms of capital available to households and individuals (factor 1), ultimately shaping nutrition outcomes.

 These three levels of the ‘basic determinants’ of nutrition, are illustrated in Figure, for clearer conceptual understanding and practical application. The left-hand side of Figure illustrates how deeply normative issues of power and equity affect structural trends and institutional distortions to condition the resources available to different groups for accessing food, health and care. When assessing the basic determinants therefore, children are affected through both the attributes of their families and the norms of their societies, and individual nutrition outcomes are determined by the interplay of these fundamental issues within different contexts.

**Figure:**
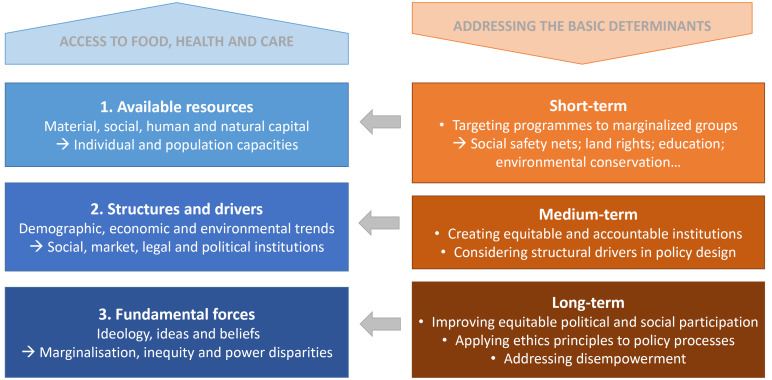
Framework for Understanding and Addressing the Basic Determinants of Nutrition.

## Examples of the Basic Determinants in Action

While child stunting is reducing steadily at the global scale, different countries and different population groups within countries are experiencing much slower declines which are slowing progress overall; and other important outcomes such as hunger and obesity are rising, often in these same groups.^24^ What sets these groups apart is their experience of the basic determinants described above. While all forms of malnutrition are underpinned by the same conceptual determinants,^4^ who is marginalised and how they experience these determinants will change with context: In Vietnam for instance, it is ethnic minority groups who are most marginalised and who continue to experience higher rates of malnutrition^25^; and in many contexts, including countries in South Asia, poor nutrition has been strongly associated with gender inequality.^26,27^

 Below, to illustrate this further, examples of the interactions between the resources, structures and fundamental forces comprising the basic determinants of nutrition are explored through existing evidence on the social determinants of health; evolving food systems; and the role of family structures. These map, respectively, to the underlying determinants in the UNICEF framework: health, food and care.

###  Health Equity and the Social Determinants of Health

The World Health Organization (WHO) Commission on the Social Determinants of Health concluded, based on its three-year examination of the global evidence, that “poor social policies, unfair economics, and bad politics are killing people on a grand scale.”^28^ It focused on the “causes of the causes” that lie beyond the proximal reasons for poor health. Those who have studied the impact of such health disparities in children have outlined just how systematic and entrenched they can be; they are therefore labelled inequitable, rather than simply unequal, as they stem from disadvantage which accrues systematically to particular groups of people because of their socio-economic position.^29^

Poverty is one such driver. Poorer children are not only more exposed to risks such as unhealthy sanitary conditions or pollution, but also more likely to have lower resistance to illness – in part due to the links between the immune system and nutrition, or suboptimal foetal development leading to low birth weights.^30^But the unfairness continues all the way through their contact with – or their very exclusion from – health systems: Poorer children are the least likely to receive preventative interventions such as vaccination, and when ill are less likely “to be taken to an appropriate healthcare provider.”^30^ If receiving healthcare, they are then “less likely to receive appropriate care because facilities serving poor communities are not as likely to have well-trained staff or to be stocked with drugs as facilities serving wealthier communities.”^30^ Ultimately “the odds are stacked against the poorest children at every one of these steps. As a result, they are more likely than their better-off peers to die in childhood.”^30^

Notably however, income poverty alone is not the sole factor in such cases of multiple deprivation^31^: Social exclusion and marginalization leading to and interacting with poverty includes discrimination based on gender, ethnicity and other social attributes which condition social status and therefore exposure to health risks and access to health systems.^28^ An example of such exclusion and marginalization acting together to limit nutritional outcomes is the exclusion of India’s scheduled tribes (ST) and scheduled castes in health and nutrition coverage. In the state of Odisha, for example, recent research highlights how rates of stunting amongst ST communities are at 46.1%, compared to 25.3% in non-ST populations.^32^ Coverage estimates of essential health and nutrition interventions in predominantly tribal districts show a significantly lower level of service provision than in non-tribal districts^[1]^.^33,34^ Such poor coverage may be a result of geographical marginalisation interacting with poverty and ethnic marginalisation (ST populations tend to live in less accessible hilly areas, further from road networks); though this in turn will interact with social stigma, with non-ST health providers reportedly less willing to provide services or visit the homes, hamlets or villages of ST clients.^35^

While this all paints a depressing picture, work on health equity has also charted a number of ways that communities, cities and countries have worked to tackle these basic determinants in systematic and structured ways. More generally, it is true that health system improvements tend to accrue to the richest segments of the population in proportion to their wealth (the social ‘gradient’) unless specific measures are taken to make such health systems pro-poor.^36^ But where governments have attempted to focus on the needs of poorer populations or deprived areas, in a sustained and considered way, then health equity has improved. As China, Costa Rica, Cuba, Kerala and Sri Lanka have shown, this can happen under very different political systems and circumstances, so long as political will and policy is oriented towards such equitable measures.^37^ Other sector-specific policies (in eg, social protection and education) can similarly be targeted to reach marginalized communities, some of which may be linked to health service provision including nutrition advice.^38^

###  Evolving Food Systems 

Judging whether modern food systems are effective depends on the yardstick used, but by most measures they are not working well for all: both the numbers of obese children and those that are hungry are rising, suggesting failures in apportioning entitlements to food^23^; and the numbers of stunted children and those with micronutrient deficiencies remain high, suggesting inadequate quality of food for many.^39-41^ Measured against the realisation of a right to good food, or simply consistent access to adequate food, modern food systems are not providing quality diets and good nutrition to all.^42^

A range of structural changes interact in shaping the current food system on both production and consumption sides. On the production side, food systems are moving further away from local food production for local consumption, and towards globalized supply and trade models. Such trends were well established under successive global ‘food regimes’^43^ established in the colonial and post-war periods, with global flows of capital (subsidies, land acquisition, agricultural inputs and agri-food commodity trade) central to global geopolitics, whether in the colonial flows of basic foods into imperial metropolitan centres; or in the continuing role of subsidy regimes in the United States and the European Union (EU) in supporting heavy concentration in production of staples and animal feeds such as corn, wheat, soy and dairy.^16,43-45^ Such geopolitics shaped food production not only in the colonial/high income centres of power, but fundamentally shaped world markets in the shape of flows of highly subsidised soy from the United States, or similarly ‘dumped’ grain surpluses from the EU, aided by tariff walls, export subsidies and ‘food aid’ policies.^43^ Collectively, these geopolitical considerations have set the terms of trade for both diets and the agricultural livelihoods on which a large proportion of the world’s poor still depend, as well as binding narratives on what is possible in terms of dietary or food system change.^46^

Agriculture and food products were included in world trade agreements from the time of the General Agreement on Tariffs and Trade agreement in 1994, opening markets to business forces that had previously been state-controlled with a food security focus, further shaping food environments in terms of availability, price and marketing of foods, mostly with the effect of limiting production possibilities for Southern producers facing the unbeatable combination of growing commercial size, vertical integration and heavily subsidised or protected crops grown in the North.^10,47^ The globalization of the food system, accelerated since that time, has led to bottlenecks concentrating power in an ‘hourglass’ shape with millions of producers and consumers at either end, but only a few large processors and marketers in the middle; in many of these middle stages, only 4 firms control 40% of the food market, for instance.^48^ The power to make food decisions is therefore moving further away from producers and consumers (and even from national governments), with national policy space increasingly limited or bound by multilateral trade rules or multilateral/bilateral investor protection treaties, or associated ‘regulatory chill’^49^ Newer agricultural powerhouses such as Brazil now join existing powerful agri-exporting nations and groupings such as the United States and the EU in shaping subsidy, investment and global trade regimes in the interests of their export industries^47^; meaning both national policy and outward looking trade policy is increasingly also shaped by ‘regulatory capture’ by various commodity lobbies or food based commercial interests.^50,51^ On the consumption side, economic and in some cases ecological forces or shocks are moving more people to urban areas and towards different food acquisition strategies; and socio-cultural forces and marketing are changing preferences and food aspirations.^52,53^ There have been positive and negative outcomes of these structural changes for food and nutrition, but the culmination in much of the world is a ‘nutrition transition’ away from traditional diets towards a similar global dietary pattern.^10,52^ Which foods people can access in this changing environment depends largely on their resources, with food price changes affecting the poor the most because they spend a higher proportion of their income on food.^10^ Notably, those with financial resources can in general access the positive fruits of food system change in the form of diverse nutritious and fresh foods, while poverty – built on marginalisation – restricts these choices to the basic (staple foods) or the cheap (long-life processed foods).^54,55^

In addition to these large global changes, local forms of marginalization beyond poverty also affect food access, for instance hunger tends to be higher in countries with limited or contested rights to land and water for some groups,^10^ and the intersection of existing malnutrition (limiting labour and learning) with other forms of inequity amplifies disparities.^56^ One of the most economically poor and environmentally fragile parts of rural Bangladesh – the wetland Haor communities in the country’s North East – provides an important localised example of how multiple aspects of marginalization and structural change interact. Here physical, social, cultural and natural capital combine in the form of land and fishing rights only available to traditional rights holders^57^or beneficiaries of new resource sharing arrangements. Such factors are compounded by beliefs around gender – women are both less likely to be traditional rights holders or beneficiaries of new schemes, and generally have a very low level of participation in decision-making – factors which combine with low health and reproductive service access to lead to larger families and intra-household pressures on available food.^57^ The region is also home to extremely poor migrants from others parts of Bangladesh or surrounding countries, attracted by the promise of marginal land. The situation of the Haor illustrates, therefore, how the extreme poor, particularly migrants and women, are excluded from natural capital essential to producing or accessing food, if they lack the physical or social capital of family land rights (a form of social institutions); or the social and cultural capital necessary to access new resource management programmes (a political institution) which tend to privilege longstanding residents of the region, existing large landholders, and men.^57-59^ All these factors combined go some way to explaining why communities surveyed in the Haor region had a stunting rate of around 45% in 2016,^60^ much higher than the Bangladesh average at that time^[2]^.^61^

Ultimately, differences in food access and nutrition outcomes are demonstrably avoidable, and hence inequitable; social justice principles demand they be addressed.^10^ Some food access issues can be addressed through policies in agriculture, land, trade and social protection – though individualized food security and nutrition programmes at a local level, such as developing people’s cooking skills and nutrition education, can potentially de-emphasise the socio-political contexts that structure unequal nutrition outcomes in the first place.^62^ But food security is not just a policy issue: eating behaviours are a response to daily living conditions, so change can be sought directly through the food system, but also through broader political, economic, social and cultural pathways.^10^

Food systems are also about where power sits, and conceptual tools are available to identify the different forms of power that exist at different levels within the food system, which then allow interventions to focus on where power is exerted, and allow activists to exploit spaces for change.^56,63^ Changing definitions can also allow different actions, for instance the concept of food sovereignty has a broader vision than the accepted definition of food security, including ideas around community rights and power to locally manage food system resources and trade, explicitly taking into consideration concentration of power in the current global food system.^62^ Sustainably and fairly improving food access requires addressing unequal distribution at its root – addressing issues such as poverty and land access – and therefore requires revealing and challenging power disparities at multiple levels, from addressing power among food system actors to empowering marginalized groups in society.^10^

###  Gender, Family Structure and Childcare

Every household has to make decisions on how to deal with balancing income-generating and productive labour, with care and reproductive labour (the unpaid work done as part of child-care or care for other family members and the home environment). These decisions on the division of household labour are seen to take account of both practical factors (such as time availability of household members, and the relative resources of men and women in the household) and ideology and broader social norms (such as patriarchy and gender roles).^64^ The relative weight of these different factors depends on context, and these decisions will therefore be taken differently in different social and economic contexts over time.

A feature of society in the Organisation for Economic Co-operation and Development (OECD) countries over the past century for example has been a remarkable rise in female labour force participation and closing of gender-based education and pay gaps, alongside declines in fertility rates and declining household size.^65^ In these countries, labour participation has been linked to economic change (such as industrialization), social and political change (such as women’s movements in the 1900s and 1960s), and demographic change (such as debates around fertility and population size).^66^ The picture is different in other contexts, with less than 30% of women documented as participating in the labour force in the Middle East, North Africa and South Asia.^67^

Notable in most social research on household labour roles in these very different contexts is that women continue to provide a majority of care and reproductive labour, whether employed or not.^64,68^ Over different locations and times, women have juggled multiple roles inside and outside the home. Family structure and childcare as a determinant of nutrition therefore reference the position of women in families and societies, and the power of young women of childbearing age relative to men and to women who are older or of a higher social class. It has long been recognized that women with greater status by various measures tend to have more control of resources, better access to information, and better self-confidence, self-esteem and mental health; and that their children tend to have better nutrition, as do women themselves.^69^ A common but poorly evidenced narrative is that women, particularly in poor households, must undertake productive work to survive, and so have less time for ‘reproductive work,’ which may lead to poorer child nutrition outcomes; while non-poor households are less sensitive to this trade-off.^70^ Responses to time pressure – and therefore effects on nutrition outcomes – have, however, been found to differ according to household contexts beyond income, such as household composition and the availability of other household members to take on care burdens^71^ – particularly, in some contexts, kin we might not normally think of such as grandmothers and siblings.^72^ Moreover, in some contexts, such as many OECD countries, child outcomes have improved even as women have worked more outside of the home; though this is not to say there are never trade-offs between economic labour and childcare, with kin not always available in some urban contexts, such as was found in one study in Kenyan cities.^73^

The difference is not, therefore, in whether women work, but in how they are supported in their multiple roles by their family, employers, society and government, as households’ social and economic contexts change due to structural economic and social drivers. Enabling environments for the care of young children remove or remedy structural and social barriers. A much-studied example is the enabling environment for breastfeeding as a key battleground over women’s rights and responsibilities in nutrition. Where women are not permitted to enter the workforce and are expected to play a purely reproductive role, or where they must work to survive at the expense of childcare, there are evident ethical issues of choice which policy can play a role in overcoming. In Vietnam for example, a key advocacy focus over the past ten years has been for an expanded maternity leave policy, to provide women working in the formal sector with a minimum of six months paid maternity leave which equals the six months of exclusive breastfeeding suggested in international recommendations.^74^ In 2012, Vietnam’s National Assembly approved an increase of maternity leave from four to six months, in addition to an extension of a current ban on the advertising of breast milk substitutes from 6 to 24 months; both policies were intended to protect and promote breastfeeding in the country’s expanding female workforce, though women working in informal sectors and traditional agriculture are still not protected.

Where employment is desired or required, good work and higher wages mitigate the negative effects of lower breastfeeding on nutrition outcomes^75^: despite significant changes in family structures, child outcomes such as mortality have improved in OECD countries over time despite reductions in breastfeeding.^65^ Most positively, parental policies supporting both productive and reproductive roles are present in many higher-income countries, aimed at enabling women (or families) to combine or choose between career and parenthood, and altering social norms regarding gender roles^66^; these maternity protections are not present in many low- and middle-income countries where women are entering the labour force, however, or in more rigidly market-based economies such as the United States. Policy options to support breastfeeding (either alongside or instead of work, depending on choice and circumstance), include maternity leave policies, health insurance for lactation support, regulations to restrict the marketing of breast milk substitutes, and baby-friendly hospitals.^76^ Beyond specific policy options, it has been noted that “to overcome the gender bias that is deeply entrenched in systems of social protection and to make citizenship truly inclusive, care must become a dimension of citizenship with rights that are equal to those that are attached to employment.”^77^

## Addressing the Basic Determinants

Drawing on the framework outlined in this paper (Figure) it follows that the basic determinants of nutrition can be addressed at different levels (the right-hand side of the figure): intervening directly in the resources available to different groups in the short term (including social safety nets, land rights, education and conservation); considering how institutional and structural forces are affecting the underlying determinants in different contexts in the medium term; and working more deeply on issues of marginalization, equity and power over the long term (expanded below).

###  Individual Human Rights and Collective Commons Frameworks

Urban Jonsson, an original architect of the UNICEF nutrition framework, speculated in 2010 that two narratives of nutrition were in competition: the ‘investment in nutrition paradigm,’ proposing that allocating more money to technical nutrition interventions to scale up service provision to larger populations would speed improvements in malnutrition outcomes; and the ‘human rights approach to nutrition paradigm,’ proposing that political action towards greater entitlements to and accountability for good nutrition would be required to catalyse improvements in both process and outcomes.^78^ Rights to food^42,79^ and health,^80^ as well as women’s rights^81^ and the rights of other marginalised groups,^82^ emerged in much of the literature we reviewed for this paper, and speak directly to the issues of marginalisation and the basic determinants of malnutrition discussed above. While the international nutrition policy community has made huge strides in framing nutrition as an investable technical field, the potential of a human rights approach to nutrition is much-invoked but less explored. No international human rights covenant explicitly recognizes the right to nutrition.^83^ Much of the discussion of a right to nutrition in international development debates has been incorporated into a discussion of the right to food – defined as the ability of people to feed themselves with dignity with foods that are available, accessible and adequate.^86^ Based on the framework of international human rights law and the values that underpin it, core principles of a rights-based approach in practice are participation, accountability and non-discrimination^84^ – direct corollaries to the marginalisation and disempowerment inherent in the basic determinants of malnutrition. Some food and nutrition programmes internationally have applied a selection of rights principles by, for example, targeting the most nutritionally-vulnerable groups, analysing the underlying causes of hunger, promoting participation and empowerment, and undertaking rights-focused evaluations.^85^ It has been suggested however that other critical elements of rights-based approaches are frequently overlooked in practice, particularly understanding stakeholders’ roles and obligations, integrating legal aspects into programmes, incorporating rights into monitoring systems, and integrating recourse and claims mechanisms into accountability programmes.^85^ What remains – if a human rights based approach is desired – is to arrange these pieces of the puzzle more squarely within existing human rights frameworks in order to acknowledge these as entitlements and duties rather than passive receipt of inputs or development programmes, and completing the list of factors that comprise a human rights framing, such as equity considerations (reviewed in sections above); accountability and participation (below); and a focus on informing the claims of rights-holders and improving the capacity of duty-bearers to respond. It has been suggested that there is no clear norm for implementing rights-based approaches to hunger^86^ or malnutrition,^87^ which may explain in part why human rights have not become a dominant paradigm for nutrition action.

Critiques of human rights suggest that they are overly-individualistic and focused on Northern ontologies.^88^ Deriving from equally vibrant and related debates, the idea of food as a commons claims that it is not “feasible to reach the right to adequate food (an entitlement) and food and nutrition security (a Global Public Good) by means of food as a commodity (a for-profit private good) under conditions of extreme inequality” generated by the basic determinants described above.^89^ Acts of ‘commoning’ (forms of collective production, consumption and stewardship/governance of food^90^ or health^91^ resources) as well as parallel movements focused on food sovereignty and agroecology^92-94^ have emerged alongside — and in many cases, in active resistance to — mainstream, technical and economistic framings of these determinants of malnutrition, or even the individualism implied by some rights based approaches. Despite their differences, both human rights and commons approaches speak to the need for inclusion, rather than marginalisation, of groups disadvantaged through the basic determinants of malnutrition – and therefore to participation and accountability in practice.

###  Accountability and Participation in Practice

The perspectives, preferences and interests of the malnourished, and particularly children or their carers, are rarely taken into account explicitly when designing policies and programmes in most settings, though traditions of community-based projects exist with varying degrees of active community consultation and involvement.^95-99^ Forms of redress if these policies and programmes fail to have the required impact, real ‘accountability,’ is also rare. Giving voice to communities regarding the types of services and interventions they are intended to benefit from and ensuring they can hold the system to account is a critical component of their success. But as those working on accountability and participation from within development studies disciplines have long argued, processes of accountability and participation are intensely political, contextual and complex and do not fold easily into the kinds of toolboxes and data-heavy technical exercises that are increasingly being promoted in their name in many contexts of international nutrition practice.^100^ If we are to avoid such endeavours becoming nice sounding development ‘fuzzwords,’^101^ then some real thought needs to be given to how various means of encouraging participation and accountability are linked to real political systems, contexts and local struggles. Stand-alone local-level initiatives need to be vertically integrated with national-level measures for political redress, and vice-versa.^102,103^ Attention needs to be paid to community and national dynamics of politics and power: while high levels of inequality can be seen to inhibit participation, they can also lead to new forms of collective action and resistance.^104^

Accountability and participatory approaches focused on child nutrition are not well documented in the global literature, but a few accounts exist. The region of South Asia, for example, has been a particularly rich area for experimentation and innovation in this area^105^ – and examples range from broader structural attempts to address inadequacies in services, to community-level action focused on enhanced accountability and participation. The former includes India’s Right to Food Movement, which paid particular attention to the need to ‘universalise’ and improve India’s community nutrition programme, the Integrated Child Development Services.^105,106^As well as pursuing action through India’s courts and political system and securing service provisions in the National Food Security Act, activists associated with the movement have been involved in community level ‘social audits,’ where government services are audited and examined at community public hearings. An non-governmental organization in the state of Odisha has been trialling such social audits for community nutrition and other National Food Security Act programmes, working with mothers and community health workers and bringing together communities in local village council meetings, while documenting the process for others to follow in a social audit training manual^[3]^. Importantly, their activities have focused on not only on village-level issues, but raising systemic issues at district and state levels to concerned officials.^35^

An alternative or complement to accountability for existing government service provision are participatory approaches which bring communities and health workers together in a common process of diagnosis of local problems and locally practicable solutions. While they can and should be linked to more vertical strategies of accountability and redress, they are also helpful in situations where tackling more structural supply-side issues are not immediately achievable; and they can help in addressing the knowledge-poverty facing carers of children in many poor communities due to broader issues of marginalisation, lack of education and power.^107,108^

Examples of activism at the national and global level leading to improvements in the political priority accorded to nutrition are easier to find and are becoming well documented: see for example.^109-111^ The role that civil society actors have played in bringing attention to the issue – whether through a more emotive focus on child deaths, or focusing on the hard data in terms of levels of malnutrition such as stunted child growth – has been important in most of these cases. But a range of other factors – from evidence gathering, to finding the right ways to frame and communicate issues, to governance structures – have been shown to be important.^112^ Frameworks used to assess agenda-setting and political commitment see for example^113,114^ can be useful in focusing national or global actors on the combination of strategies to improve accountability and commitment to action likely to benefit children’s nutritional status, while tools such as the Hunger and Nutrition Commitment Index can be used in conjunction with civil society activism to help reframe nutrition politically and garner action at national levels.^115^

## Conclusion

 This paper has laid out how and why basic social, economic and political factors are slowing declines in undernutrition in some populations, and driving a rapid nutrition transition in others. We have reviewed above with relation to aspects of food, health and care how the fundamental capacities and resources available to different groups interact with changing demographic, economic and political trends and institutions, producing outcomes which at their root are embedded in social norms about power and position, influenced by ideas and ideologies in different contexts. Marginalization and social exclusion shape social standing and access to resources and power, and hence ability to ride waves of structural change in accessing good nutrition.

While there is a tendency to focus on certain aspects of marginalization such as gender and wealth in nutrition research and practice, poverty and patriarchy are not the only important ‘basic determinants’ for nutrition. Forms of exclusion from essential goods, services, resources and politics can be based on a number of socio-biological or socio-spatial criteria including gender, ethnicity, age, disability, sexual orientation, or geographic location, among others.^116^ In many cases, these causes of discrimination are multiple and intersecting; they accrue over time to particular groups to become the systemic differences in life chances we tend to assume are a natural facet of poverty or malnutrition. Social epidemiologists familiar with this field have used the term ‘embodiment’ to describe “how we literally incorporate, biologically, the material and social world in which we live, from conception to death.”^117^Children’s bodies in particular become the agents via which social and material deficits are passed from one generation to another – with children born to malnourished mothers more likely to suffer birth irregularities of danger to both mother and child, be lower birthweight and be malnourished through their childhood.^5^ Such early embodied disadvantage only becomes entrenched throughout the lifecourse for the marginalized; where ill health and malnutrition leads to lost education, employment and income – or increased expenditure on healthcare treatment and emergencies – this can lead already vulnerable families into further cycles of deprivation which are hard to escape.^2^

Acknowledging the basic determinants as unnatural and avoidable systemic processes which drain particular groups of resources and power is key to understanding and addressing them.^118^ Many of the basic determinants of malnutrition affect children not as individuals but as part of families and groups that exist in specific social, economic and political contexts which condition access to the resources that enable good nutrition. In assessing and addressing the basic determinants therefore, it is important to understand the particular circumstances of different population groups and their access to programmes and services, and to social and political redress that have been well described in literatures on human rights and the collective commons, among others.

While we draw here on thinking from development studies and its allied disciplines, the basic determinants are little-studied with relation explicitly to nutrition.^119^ We suggest that more research is needed to understand how the basic determinants affect nutrition in different population groups in different contexts, drawing on work exploring these issues in other development disciplines. For nutrition practice, we suggest that policy-makers and programmers assess these basic determinants in any context analysis before action, and evaluate their contribution to differential programme impacts for different groups; and pay explicit attention to addressing marginalization and inequity underpinning power disparities in the longer term. The framework presented in this paper suggests specific areas within the basic determinants for the focus of research and practice, and frameworks such as human rights and the commons offer ideas on participation and accountability to address the basic determinants in action.

Challenges remain for the nutrition sector with regards to securing explicit acknowledgement of dimensions of power and inequity – in families, societies, political systems and food systems – that are essential in both driving and tackling the basic determinants; yet achieving this recognition is not in the interests of the powerful. Reports such as the WHO Commission on the Social Determinants of Health^28,36^ have moved such targeted approaches to the mainstream – though as adherents have pointed out, the project of health equity is a continual struggle against broader structural trends such as the global financial crisis.^80,120^

 Action on the basic determinants requires acknowledgement that local settings and ideas matter, and that there will never be a one-size-fits-all model for eliminating malnutrition. Despite this focus on the local, broad patterns can be identified and we have laid these out as the basic determinants of malnutrition; tackling these basic determinants has the potential for larger impact on nutrition that is more sustainable than solely addressing technical interventions to the underlying and immediate causes, and addressing the basic determinants is a clear ethical and development imperative itself. These basic issues are however less tractable and more difficult to address in short funding and political cycles, requiring sustained action over the longer term both from above (policy) and below (resistance). We have shown here that the basic determinants are not a black box of ‘context,’ but can be broken down into comprehensible issues that are amenable to change, and should be considered explicitly in research and action to reduce the global burden of malnutrition.

## Acknowledgements

 The authors thank Manmeet Kuar for conducting the literature review on which this paper was based.

## Ethical issues

 Not applicable.

## Competing interests

 Authors declare that they have no competing interests.

## Authors’ contributions

 JH and NN together contributed all conceptual and empirical work, and undertook all writing and editing of this manuscript.

## Funding

 Funding was provided by UNICEF as part of the State of the World’s Children Report background paper process.

## Endnotes


^[1]^ In Koraput district in Odisha, for example, only 58.4% of mothers reported receiving the recommended minimum number of 4 antenatal care visits during pregnancy, compared to 81.8% in Puri district. Likewise 67.1% of children had received a full set of immunisations in Koraput, compared to 88.2% in Puri. All data from POSHAN District Nutrition Profiles http://poshan.ifpri.info, drawing on IIPS 2016, UNICEF 2016.

^[2]^ The Bangladesh Demographic and Health Survey reported a rate of 36% in 2014 and 31% in 2018.

^[3]^ http://nirdpr.org.in/nird_docs/socialaudit/English-sa.pdf.

